# A *Mycobacterium tuberculosis *cluster demonstrating the use of genotyping in urban tuberculosis control

**DOI:** 10.1186/1471-2334-9-151

**Published:** 2009-09-08

**Authors:** Gerard de Vries, Rob AH van Hest, Conny CA Burdo, Dick van Soolingen, Jan H Richardus

**Affiliations:** 1 Department of Tuberculosis Control, Municipal Public Health Service Rotterdam-Rijnmond, P.O. Box 70032, 3000 LP Rotterdam, The Netherlands; 2 Department of Public Health, Erasmus MC, University Medical Center Rotterdam, P.O. Box 2040, 3000 CA Rotterdam, The Netherlands; 3 National Mycobacteria Reference Laboratory, National Institute of Public Health and the Environment, P.O. Box 1, 3720 BA Bilthoven, The Netherlands; 4 Division of Infectious Disease Control, Municipal Public Health Service Rotterdam-Rijnmond, P.O. Box 70032, 3000 LP Rotterdam, The Netherlands

## Abstract

**Background:**

DNA fingerprinting of *Mycobacterium tuberculosis *isolates offers better opportunities to study links between tuberculosis (TB) cases and can highlight relevant issues in urban TB control in low-endemic countries.

**Methods:**

A medium-sized molecular cluster of TB cases with identical DNA fingerprints was used for the development of a visual presentation of epidemiologic links between cases.

**Results:**

Of 32 cases, 17 (53%) were linked to the index case, and 11 (34%) to a secondary case. The remaining four (13%) could not be linked and were classified as possibly caused by the index patient. Of the 21 cases related to the index case, TB developed within one year of the index diagnosis in 11 patients (52%), within one to two years in four patients (19%), and within two to five years in six patients (29%).

**Conclusion:**

Cluster analysis underscored several issues for TB control in an urban setting, such as the recognition of the outbreak, the importance of reinfections, the impact of delayed diagnosis, the contribution of pub-related transmissions and its value for decision-making to extend contact investigations. Visualising cases in a cluster diagram was particularly useful in finding transmission locations and the similarities and links between patients.

## Background

Tuberculosis (TB) is an infectious disease that caused 9.2 million new cases and 1.7 million deaths in the world in 2006 [1]. Over one-third of the world population is infected with *Mycobacterium tuberculosis*, the causative organism of TB. The asymptomatic stage, without clinical or radiological symptoms, is referred to as latent TB infection (LTBI) and is usually determined by a tuberculin skin test (TST). Persons with LTBI have approximately a 10% lifetime risk to progress to disease.

In descriptive epidemiology, the distribution and transmission of infectious diseases is usually described in terms of place, person, and time. This approach is appropriate for diseases with a short incubation period but far less so for chronic diseases such as tuberculosis (TB), which progresses from latent infection to active disease over a period ranging from months to many years. In TB epidemiology, graphic presentation is often limited to trend description over years in a defined region, with sociodemographic characteristics of the patients [1,2]. However, in outbreak management, one would like to know which secondary cases are caused by an index patient, i.e. the source case of the outbreak. Presentation in a time line may help to detect epidemics, plan interventions, and evaluate these efforts.

In many developed countries of the world, *M. tuberculosis *isolates of TB patients are subject to DNA fingerprinting. The most widely applied method has been the restriction fragment length polymorphism (RFLP) typing. The technique is based on identifying specific insertion sequences (IS*6110*) that are common in bacterial genomes [3]. Clusters are defined as groups of patients having isolates with fully identical RFLP patterns or, if strains have fewer than five IS*6110 *copies, with identical sub-typing determined by the Polymorphic GC-Rich Sequence probe [4]. DNA fingerprinting has improved the understanding of TB transmission and helped to identify outbreaks, high-risk groups, and laboratory cross-contaminations [5,6]. To allow adequate interpretation and comparison however, molecular studies should involve a high proportion of cases in a population, be combined with conventional epidemiological investigations, and specify patient characteristics and study length [6,7]. In the Netherlands a system of universal genotyping exists for more than 14 years with nearly all *M. tuberculosis *isolates genotyped, and results are available at Municipal Public Health Services (MPHS) [8,9].

We describe a cluster of cases in the Rotterdam area, introducing a novel cluster diagram that shows epidemiologic links between cases. The cluster analysis is used to discuss relevant issues in urban TB control in low-endemic countries.

## Methods

### Setting

The MPHS Rotterdam-Rijnmond covers the municipality Rotterdam (about 600,000 inhabitants) and 27 other municipalities, serving a total population of 1.3 million. Between 1993 and 2006, 2,562 TB cases were reported, resulting in an average annual incidence rate of 23.3 per 100,000 for Rotterdam municipality and 6.6 per 100,000 for other municipalities. Of the 2,562 cases, *M. tuberculosis *was cultured in 1,969 (76.9%), of which 1,938 (98.4%) were RFLP-typed. Of these, 881 (45.5%) were unique or initially unique first cases in a cluster, and 1,057 (54.5%) were non-first cases in a cluster. Of the RFLP-clustered cases, 391 (37.0%) were the 2-5^th ^case in a national cluster, 529 (49.9%) the 6-50^th ^case, while 139 cases (13.2%) were 51^st ^or subsequent case in three large national, predominantly Rotterdam, clusters. TB control nurses of MPHSs explore possible links between RFLP-clustered cases during visits to patients.

### Study population

A medium-sized molecular cluster of 28 cases with considerable transmission in and around Rotterdam was used to develop a cluster diagram with Microsoft Visio^® ^software including a time-period bar, zones suggesting locations of transmission, and symbols representing the ethnic background, disease site, and infectious status of patients, and arrows representing documented epidemiological links between cases. The causative *M. tuberculosis *strain was susceptible to all first-line TB drugs. Four culture-negative epidemiological linked cases were included in the cluster diagram.

### Contact tracing

Contact investigation was executed according to Dutch guidelines, i.e. close contacts of smear-positive TB patients are examined in two rounds with a TST performed immediately after diagnosis of the index patient and two months after their last contact. Cases identified with a latent TB infection are generally treated with six months isoniazid. Contacts ineligible for TST (born before 1 January 1945, vaccinated with bacille Calmette-Guérin (BCG), or having positive TST or TB in the past) are examined twice with a chest radiograph: immediately after diagnosis of the index and three months after the last contact with the index. An investigation is extended from close to casual contacts on finding high rates of recent TB infections or TB cases. All those investigated are linked to the index patient in the client information system of the Dept of Tuberculosis Control. Using this data, we analyzed the results of TSTs and radiographs of contacts linked to patients in our cluster.

The Medical Ethics Committee of Erasmus MC, University Medical Center Rotterdam, Rotterdam approved the study protocol.

## Results

### Cluster description

**Figure 1:**
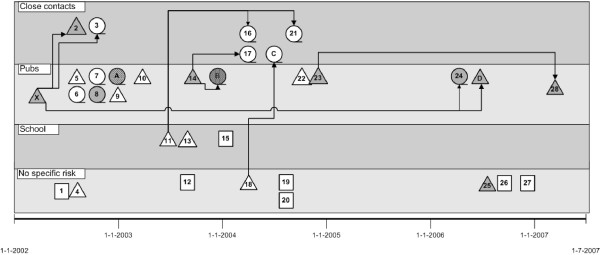
**Cluster diagram of a medium-sized molecular tuberculosis cluster**. Each symbol represents a patient. The symbols are placed on a timeline according to the month of specimen collection. Triangles denote patients with smear-positive pulmonary tuberculosis (TB), circles patients with smear-negative pulmonary TB, and squares patients with extrapulmonary TB. Grey coloured symbols indicate patients with Yugoslavian ethnicity, and for simplicity, all cases with other background have uncoloured symbols. (Origins included Netherlands in 4 persons, Surinam or Netherlands Antilles in 7, Morocco in 5, Indonesia in 3, India in 1, and Turkey in 1). Cases with a number are culture-confirmed; cases with a letter are culture-negative cases epidemiologically linked to the cluster. Lines between cases indicate that cases know each other (see also Results). Note: The diagram can be made with different coloured symbols for ethnic background. Instead of symbols, bars could be used to show the duration of symptoms of infectious TB patients.

The first strain identified in our cluster (Figure 1) was from a Surinamese person with pleural TB (case 1). One month later, pulmonary TB was diagnosed in a Bosnian woman (case 2), who four months earlier had reported to the Dept of Tuberculosis Control that a Bosnian man was her houseguest for several weeks and had subsequently entered a German hospital with smear-positive pulmonary TB (case X). Due to prior BCG vaccination, the woman had a chest X-ray at presentation and three months after her last contact with patient X. Her second radiograph showed discrete upper lobe infiltrative lesions. One month later, her sputum culture became positive for *M. tuberculosis*; at her next consultation, radiographic changes were more pronounced and sputum smears were positive.

Within a few months after clustering of cases 1 and 2, another eight cases were diagnosed with TB caused by a mycobacterial strain having an identical fingerprint. Almost all these patients frequented a certain pub in Rotterdam, and the TB control nurses found that patient X had been ill for several months and also visited this pub. Case 8 was a five-year-old child who had a positive TST and hilar lymphadenopathy on the chest X-ray. TB was diagnosed by positive culture for *M. tuberculosis *of the gastric lavage fluids. His brother (case A) likewise had a positive TST and hilar lymphadenopathy, and started TB treatment without further diagnostic investigation. Both children often accompanied their grandfather to the above-mentioned pub, which was owned by relatives.

Several months after case 10, smear-positive pulmonary TB was diagnosed in an 18-year old student of a vocational training school (case 11). He had been coughing for five months and visited his general practitioner several times. Contact tracing revealed LTBI in all his seven family members, in classmates, and persons at his vocational training site, including five persons with TST conversions. After two months, a second student from the school was diagnosed with smear-positive pulmonary TB (case 13). He did not share ethnic background or classes with case 11. His family, friends, and classmates were examined, but extension of the contact investigation was actually decided on the basis of DNA fingerprint results. When they confirmed clustering, the entire school was examined and yielded one case of pleural TB (case 15) - confirmed by culture of a pleural biopsy - and 40 LTBI cases. Disease developed in a brother of case 11 (case 16) despite treatment with isoniazid; he later admitted taking only three weeks of medication despite attending all medical check-ups. The disease developed in another relative of case 11 (case 21), who had been treated for TB twenty years earlier.

Five other patients (cases 14, 22-24, and D) had a link with the pub and were most likely infected by patient X. Patient D, one of the pub owners, had a chest X-ray in a general hospital four months before her TB diagnosis; it showed bilateral opacities and she was treated for pneumonia. Despite repeated visits to her general practitioner, smear-positive pulmonary TB was diagnosed only when she travelled to her native country. In the interim, she had stayed with her disabled child several months in a hospital, and had infected a nurse (case 27) who seven years earlier had completed LTBI treatment after converting the TST.

### Patient characteristics

The 32 patients in this outbreak (excluding case X) were aged 5 to 67 years (average 33 years); 81% were male; 25 (78%) had pulmonary TB, of which 14 were smear-positive. None were found coinfected with HIV. All 32 patients completed TB treatment. Of the 32 patients, 21 lived in Rotterdam, seven in two nearby towns, and one (case 22) resided in a village 50 km from Rotterdam. After genotyping, this last patient was reinterviewed and revealed regular visits to the Rotterdam pub up to one year before diagnosis. The remaining three (cases 4, 18 and 27) lived outside the Rotterdam area but had links to the city or to other patients in the cluster.

### Second and third generation cases in the cluster

Of 32 cases, 17 (53%) were epidemiologically linked to case X (i.e. secondary cases) based on time intervals, personal contacts, or meeting places (Table 1), and 11 cases (34%) were certainly or possibly linked to a secondary case (i.e. tertiary cases). The remaining four patients (13%) could not be linked to a cluster case and were classified as possibly infected by case X (i.e. secondary cases).

**Table 1:** Parameters (time, person, place) by which cases in the cluster were linked.

Tuberculosis case in cluster	Linked to source patient	Time	Person	Place
2, 3	Case X	Yes	Yes	Yes

1, 4, 11	Case X	Yes	No	No

5, 6, 7, 8, 9, 10, A	Case X	Yes	No	Yes

14, 22, 23	Case X	No	No	Yes

24, D	Case X	No	Yes	Yes

12, 13, 15	Case 11	Yes	No	Yes

16, 21	Case 11	Yes	Yes	Yes

B, 17	Case 14	Yes	Yes	Yes

C	Case 18	Yes	Yes	Yes

26, 27	Case D	Yes	No	Yes

28	Case 23	No	Yes	Yes

18, 19, 20, 25	No link established	No	No	No

Cases with a number are culture-confirmed; cases with a letter are culture-negative cases epidemiologically linked to the cluster.

**Figure 2:**
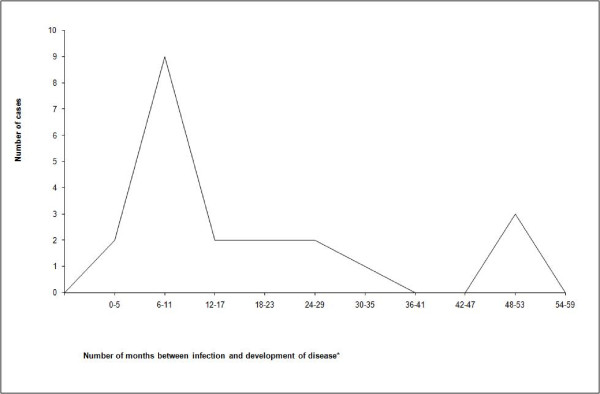
**Curve of the time between tuberculosis infection and development of disease in 21 secondary cases**. * The date of development of disease was defined as the date of specimen collection and time of infection was assumed as the diagnosis date of the index patient.

Figure 2 shows the interval between the month of case X diagnosis and the month specimens were collected from the 21 persons who were certainly or possibly infected by him. Of these, TB developed within a year of the index diagnosis in 11 (52%), within one to two years in four (19%), and within two to five years in six patients (29%).

### Contact investigation

Contact tracing was mainly limited to close contacts of pulmonary TB cases (Table 2). Three investigations were extended to many casual contacts after DNA fingerprinting confirmed that transmission had occurred, respectively, in the above-mentioned pub, vocational school, and hospital. Only a few pub visitors outside the known contacts could be tested due to non-cooperation of the primary pub owner.

**Table 2:** Contact tracing results of smear-positive pulmonary and other tuberculosis patients of the cluster.

	Number of contacts examined	Number of contacts examined with TST	Number of tuberculosis infections	Proportion of tuberculosis infections among those tested	Tuberculosis cases
Mr. X	3	1	1	100	Case 2

Case 2	23	14	1	7	Case 3

Case 4	0	0	0	0	

Case 9	24	5	0	0	

Case 10	50	41	1	2	

Case 11	194	154	24	16	(Case 16)

Case 13	45	33	0	0	

School	1,730	964	40	4	Case 15

Case 14	1	0	0	0	Case B

Case 18	11	4	0	0	Case C

Case 22	31	15	1	7	

Case 23	4	4	1	25	

Case D	3	3	3	100	

Case 25	10	2	0	0	

Hospital	100	92	1	0	

Case 28	32	8	3	38	

Other	67	32	3	9	Case A

Total	2,328	1,372	79	5.8	6

Cases with a number are culture-confirmed; cases with a letter are culture-negative cases epidemiologically linked to the cluster. Case 16 (in brackets) was diagnosed with latent TB infection and later developed TB (see also Results).

## Discussion

In retrospect, the index patient of the cluster was highly infectious, causing 11 secondary TB cases within one year after exposure. Contact investigation after detailed interviewing of an infectious patient normally identifies contacts for screening and preventive treatment. In this case, however, the index patient was initially unknown and the natural history of the disease was unimpeded. Five years after the index diagnosis, 28 culture-confirmed cases with identical fingerprints had been identified, forming a new molecular cluster. The cluster diagram highlights several issues relevant to urban TB control in low-incidence countries.

### Natural history of tuberculosis

The classification of patients in the cluster allowed us to draw a curve of secondary cases. Of these cases, TB developed within one year of infection in 11 (52%), within two years in 15 (71%), and between two to five years in six patients (29%). The curve recalls results of previous tuberculin conversion studies, which estimated that TB develops within five years in 14% of adults infected, with 60% of cases presenting within one year, 85% within two years, and 15% within two to five years [10]. Some of our late-secondary cases may have been misclassified as caused by the index patient, raising the proportion of secondary cases that occurred within two years.

The number of secondary cases reported in the first year allows one to estimate the total number of persons infected by the index patient and to predict the number to be expected in the next few years. As illustrated by our cluster, secondary cases can generate tertiary cases, and together with late-secondary cases they can protract an TB outbreak over many years [3,11-14].

Our study included four culture-negative TB cases and 75 LTBI cases, indicating that conventional epidemiological investigation and molecular typing should be combined [5]. Studies using only genotyping data exclude such cases and may thus underestimate the true extent of an outbreak and the value of active case finding.

### Reinfection

Cluster analysis and conventional epidemiological investigation revealed two reinfections (cases 21 and 27). Molecular studies have demonstrated that the extent of exogenous reinfection in recurrent TB depends largely on the epidemiological context [4,15]. In populations at low risk, most recurrence results from reactivation, although the proportion due to reinfection in some low-endemic countries may exceed expectations, ranging between 16% and 44% [16-18]. Our two reinfection cases illustrate that a contact investigation must also look carefully at previously infected persons.

### Geographical clustering and urban spread

The outbreak was mainly limited to the Rotterdam area; patients outside the area were epidemiologically linked with a clustered case. Our experience with DNA cluster analysis is that most clustered patients in the Netherlands are residents of the same geographical region.

Genotyping can be used to monitor the magnitude of the problem of TB transmission. Several studies have demonstrated that the proportion of clustered cases in urban areas comprise 20 to more than 50 percent of all local TB cases, indicating recent transmission [3,4,19-23]. A recent outbreak of an isoniazid-resistant *M. tuberculosis *strain with more than 132 cases in London and a molecular cluster of 150 in the Netherlands (of which 85% are in Rotterdam), underscore the need in large cities for interventions tailored to specific risk groups [12,13,24].

### Tuberculosis among foreign-born persons

Typically, foreign-born residents account for more than 50% of TB cases reported in low-endemic countries [2]. Although most are probably infected in their native countries [25], recent transmission in the country of residence can contribute significantly to high incidence in the foreign-born. In our cluster, the index patient directly and indirectly caused disease in 32 persons, of whom 10 (31%) shared his ethnic background and 22 (69%) did not. In general, transmission between foreign nationalities and to the indigenous nationality varies widely in low-endemic countries, probably reflecting different social mixing patterns [25-28].

### Transmission in high-risk settings

Our cluster diagram shows that half of the cases were infected in the same pub. Bars are known for high transmission rates due to crowding and poor ventilation [11,29,30]. Smoking may also increase the risk of *M. tuberculosis *infection [31]. Contact investigation is often hampered by pub owners' reluctance to assist in informing the clientele, difficulties in finding contacts, and unwillingness of infected persons to start a potentially hepatotoxic preventive treatment that precludes the use of alcohol [11].

Our cluster analysis also confirmed TB transmission at a school and in a hospital. The increased risk for health care workers is well-documented and is usually related to delayed diagnosis of TB patients [32,33]. In our study, a patient's relative was the source case of TB transmission to a health care worker, so the nosocomial setting was incidental.

### Promoting early case detection and maintaining clinical expertise

Delays in seeking medical attention or receiving diagnosis facilitate TB transmission [4]. Our index patient severely delayed seeking care despite the urging of his friends. On the other hand, two secondary patients repeatedly visited their general practitioners before being diagnosed with TB, and one of these was diagnosed abroad. Delays in these three cases led to several LTBI and TB cases. In many low-incidence countries, TB expertise is declining in general practitioners, clinical specialists, and public health professionals, but resulting delays in diagnosis can be reduced by continuing education [34]. Delays in seeking care can be reduced by public health education and low-threshold access to TB diagnostic and therapeutic services.

### Outbreak management and contact investigation

In our outbreak, 6 TB cases and 75 persons with LTBI were traced. Most LTBI cases were diagnosed in the school investigation, and treatment controlled the situation because no more cases were identified in three years of follow-up. The importance of preventive therapy was highlighted by a noncompliant patient with LTBI who subsequently developed TB.

Failure to identify and examine eligible contacts, as occurred in the pub investigation, is a major reason for disease development [14]. Our diagram shows that even detailed interviewing failed to disclose all links between patients and potential locations of transmission, a problem also encountered by others [3,4,7,11,14,35]. However, our study clearly demonstrates that clustering of cases in time and place, with similar sociodemographic patterns, resulted from recent transmission in the Netherlands.

## Conclusion

In an urban area in a low-endemic country with ongoing TB transmission, cluster analysis was a useful complement to conventional epidemiological investigation. It revealed that foreign-born patients were infected in this low-endemic country and not in their native countries, as might have been assumed. Visualising cases in a cluster diagram was particularly useful in finding transmission locations and the similarities and links between patients. It also helped to monitor the cluster and outbreak over time and to estimate the number and timing of future cases.

## Competing interests

The authors declare that they have no competing interests.

## Authors' contributions

GV was responsible for study design, data collection, data analysis, drafting and revising the manuscript, RH contributed to study design, data collection, data analysis, drafting and revising the manuscript, CB contributed to data collection, data analysis, drafting and revising the manuscript, DS contributed to drafting and revising the manuscript and JR contributed to study design, drafting and revising the manuscript. All authers read and approved the final manuscript.

## Pre-publication history

The pre-publication history for this paper can be accessed here:

http://www.biomedcentral.com/1471-2334/9/151/prepub
